# Association between oral health and incidence of pneumonia: a population-based cohort study from Korea

**DOI:** 10.1038/s41598-020-66312-2

**Published:** 2020-06-12

**Authors:** Minkook Son, Sangyong Jo, Ji Sung Lee, Dong Hyun Lee

**Affiliations:** 1 Department of Biomedical Science and Engineering, Gwangju Institute of Science and Technology, Gwangju, Korea; 2 Department of Internal Medicine, Air Force 3rd Flying Training Wing, Sacheon, Korea; 3 Clinical Research Center, Asan Medical Center, Ulsan University College of Medicine, Seoul, Korea; 4 Departement of Pulmonology and Intensive Care Medicine, Dong-A University, College of Medicine, Busan, Korea

**Keywords:** Diseases, Health care, Medical research

## Abstract

Pneumonia is related to oral health of the elderly and intensive care unit patients. However, studies on the relationship between overall oral health and pneumonia in the general population have been limited. The purpose of this study was to investigate the association between oral health and pneumonia using a nationwide population-based Korean cohort database. Data from 122,251 participants who underwent health screening and oral examinations in 2004 or 2005 were analyzed. Cox proportional hazard regression analysis was performed to evaluate the association between oral health and pneumonia. The risk of pneumonia increased significantly in groups with a higher number of dental caries and missing teeth, with respective adjusted hazard ratios (HRs) and 95% confidence interval (CI) of 1.265 (1.086–1.473; p = 0.0025) and 1.218 (1.113–1.332; p < 0.0001), and decreased significantly in frequent tooth brushing and regular professional dental cleaning groups, with respective adjusted HRs and 95% CI of 0.853 (0.786–0.926; p = 0.0001) and 0.920 (0.855–0.990; p = 0.0255). In addition, regardless of age and comorbidities, oral health status and oral hygiene behaviors were associated with pneumonia. The results indicate that improved oral health may reduce the risk of pneumonia in the general population.

## Introduction

Pneumonia is a debilitating disease that can result in death in elderly individuals^1^ and has been reported to be related to oral health^2^. The oral cavity is a complex environment for multiple microorganisms and cytokines^3^. In particular, aspiration of microorganisms and biological mediators such as cytokines and hydrolytic enzymes from the oral cavity can provoke inflammation and cause infections^4,5^. The association between pneumonia and oral health has been examined in nursing homes, and the incidence of pneumonia has been reported to be lower in elderly populations receiving oral care^6^. Besides, several studies have suggested that hospitalized intensive care unit (ICU) patients appear to benefit from daily oral cleansing^7,8^.

Oral hygiene can be improved through individual-based interventions^9^. Daily personal oral hygiene behaviors such as tooth brushing are fundamental in the prevention of periodontal disease and bacterial plaque, which can respectively lead to tooth loss and dental caries^10,11^. In addition, professional dental cleaning has been proven to reduce periodontal disease, dental caries, and tooth loss^12^. Dentists recommend tooth brushing at least twice daily as well as regular dental visits for professional dental cleaning^13^.

While the link between pneumonia and oral health has been gaining increased attention, studies evaluating the relationship between overall oral health and pneumonia in the general population have been limited. The purpose of this study was to investigate the association between oral health and pneumonia using a nationwide population-based Korean cohort from the National Health Insurance Service-Health Screening (NHIS-HealS) database.

## Methods

### Data source

This study used the NHIS-HealS database^14^. The NHIS database is registered with 97% of all Koreans and includes all insurance claims data. We analyzed the cohort data, which was extracted from a random sampling of approximately 10% of the 5 million people (age ≥40 years) who underwent health screening in 2002 or 2003. Information about demographic data (age, sex, and socioeconomics), clinical data (medical service use, disease diagnosis, and treatment), and health screening data (physical examination, laboratory tests, and questionnaires on lifestyle and medical histories) is included in the NHIS database accessible to researchers. Registrants in the NHIS database are recommended to receive a standardized health screening at least every 2 years. A detailed description of the data can be found on the relevant website^14,15^.

### Study population

**Figure 1:**
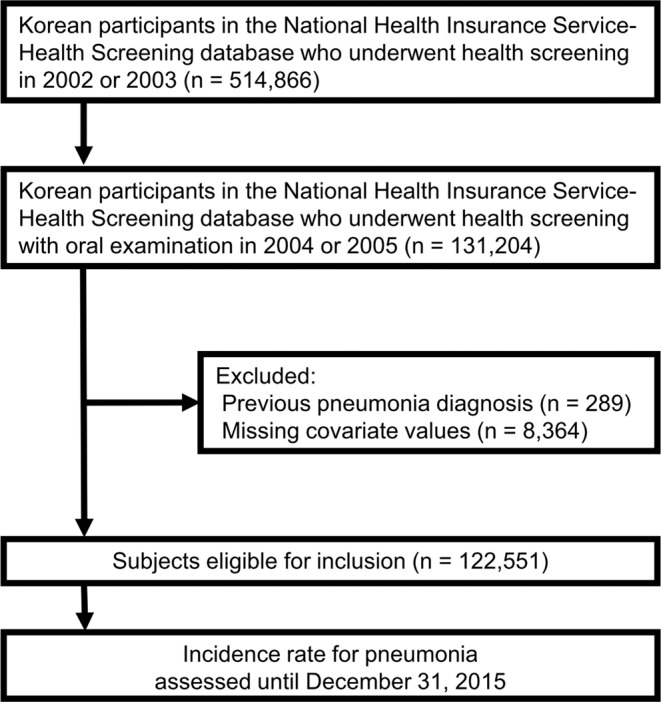
Flow of study participants.

The first two years (2002–2003) were considered as a washout period. Among the 514,866 people in the NHIS-HealS database, the participants included in this study were limited to those who underwent health screening with oral examinations in 2004 or 2005. We excluded participants with a pneumonia diagnosis before the day of health screening. Participants with one or more missing values were excluded. Finally, 122,251 participants were included in our study (Fig. 1).

### Oral health status and oral hygiene behaviors

The oral health screening program was provided to all registrants. The program was composed of oral examinations conducted by professional dentists and self-reported questionnaires. Registrants were inspected by dentists for periodontal status and for the number of decayed and missing teeth. In this study, periodontal disease was categorized as either absent or present. The number of dental caries and missing teeth was categorized as 0, 1–4, and ≥5. Oral health questionnaires included dental visits, dental symptoms, and oral hygiene behaviors. Based on the questionnaires, gum bleeding was classified as absent or present. The number of tooth brushing sessions per day was classified as 0–1, 2, and ≥3. Professional dental cleaning was classified as <1 or ≥1 time per year.

### Outcomes

The main study outcome was the incidence of pneumonia^16,17^. We used 3 definitions for pneumonia (Supplementary Fig. S1). The first definition of pneumonia (Def. 1) was defined based on the 10^th^ revision of the International Statistical Classification of Diseases and Related Health Problems (ICD-10) codes for pneumonia (J10-J18) with any prescription of antibiotics (J01). In addition to Def. 1, the second definition of pneumonia (Def. 2) includes hospitalization for 2 days or more, and the third definition of pneumonia (Def. 3) includes hospitalization for 7 days or more. The study participants were followed up on from the day of health screening at index year to the day of incidence of pneumonia, the day of death, or December 31, 2015, whichever came first (Supplementary Fig. S2).

### Covariates

Age, sex, body mass index (BMI), hypertension, diabetes, dyslipidemia, Charlson comorbidity index (CCI) category, smoking, drinking, exercise, income, periodontal disease, dental caries, missing teeth, gum bleeding, tooth brushing, and professional dental cleaning were considered as covariates. BMI was calculated by dividing body weight (kg) by the square of the height in meters (m^2^). Hypertension, diabetes mellitus, and dyslipidemia were defined using criteria from previous studies^18–20^. The presence of hypertension was defined according to the following criteria: (1) ICD-10 codes for hypertension (I10, I11) with at least one claim per year for prescription of an antihypertensive drug, or (2) systolic blood pressure ≥ 140 mmHg or diastolic blood pressure ≥90 mmHg. The presence of diabetes was defined according to the following criteria: (1) ICD-10 codes for diabetes (E10-E14) with at least one claim per year for the prescription of an anti-diabetic drug, or (2) fasting glucose level ≥ 126 mg/dL. The presence of dyslipidemia was defined per the following criteria: (1) ICD-10 codes (E78) for dyslipidemia with at least one claim per year for prescription of lipid-lowering agents or (2) total cholesterol level ≥240 mg/dL. The CCI was calculated based on preexisting diseases, including myocardial infarction, congestive heart failure, peripheral vascular disease, cerebrovascular disease, dementia, chronic pulmonary disease, connective tissue disease, peptic ulcer, mild liver disease, diabetes with and without complications, paraplegia or hemiplegia, renal disease, any or metastatic cancer, moderate or severe liver disease, and acquired immune deficiency syndrome before the start of the follow-up period^21,22^. Information on smoking status, drinking status, and exercise status was obtained using questionnaires. For smoking status, participants were classified as non-smokers, ex-smokers, or smokers. To identify drinking status, participants were categorized into non-drinking or drinking groups. Regular exercise was defined as at least five exercise sessions per week. Income was divided into two groups based on the tenth percentile in the income distribution.

### Statistical analysis

Baseline characteristics were presented as mean with standard deviation for continuous variables except age and as number with percentage (%) for categorical variables. Ages were presented as medians with interquartile ranges. The incidence rate of pneumonia was calculated by dividing the number of pneumonia cases by the total follow-up duration (person-years). The graphs for the incidence rate of pneumonia according to the oral health and oral hygiene behaviors were calculated using Kaplan-Meier curves and evaluated by the log-rank test and Gray’s test for competing risk^23^. The hazard ratio (HR) and 95% confidence interval (CI) for the incidence of pneumonia were evaluated using the Cox proportional hazards model. The proportional hazard assumption was evaluated by the Schoenfeld residuals test with the logarithm of cumulative hazards function based on Kaplan-Meier estimates. There was no interference with the assumption of proportional hazard risk over time. The multivariable-adjusted cox proportional hazard models were adjusted for age, sex, BMI, hypertension, diabetes, dyslipidemia, CCI category, smoking, drinking, exercise, income, periodontal disease, dental caries, missing teeth, gum bleeding, tooth brushing, and professional dental cleaning. Besdies, the Fine and Gray model for competing risk with death was applied to evaluate the HR and 95% CI for incidence of pneumonia^23^. Subgroup analyses according to age and comorbidities were performed. In the subgroup analysis, ages were divided into under and over 60 years categories. The presence of comorbidities was defined as the case where CCI was not 0. Statistical analyses were performed using SAS version 9.4 (SAS Institute Inc., Cary, NC, USA) and all figures were produced using R 3.6.0 (https://www.r-project.org/). A two-sided p-value < 0.05 was considered statistically significant.

### Ethical consideration

This study was approved by the Institutional Review Board of the Dong-A University Hospital (DAUHIRB-EXP-20-006), which waived the requirement for informed consent in regard to the anonymized data analyzed retrospectively.

## Results

### Baseline characteristics

The study population with Def. 3 comprised 122,551 healthy participants with no history of pneumonia (Table 1). The mean age of participants was 52.4 years and 64.0% were male. The mean systolic blood pressure and fasting glucose were 125.5 mmHg and 96.7 mg/dL, respectively. 49.6% of participants had periodontal disease. 2.7% and 6.0% of participants had five or more dental caries and missing teeth, respectively. According to the self-reported questionnaires, 45.0% of participants brushed their teeth three times or more per day, and 26.0% replied having professional dental cleaning at least once per year. Supplementary Tables S1–S2 show the baseline characteristics of the study participants according to the definition of pneumonia. Supplementary Tables S3–S7 compare the baseline characteristics of participants according to oral health status and oral hygiene behaviors in Def. 3 of pneumonia.

**Table 1:** Baseline characteristics of study participants (with Def. 3).

Characteristics	Total (n = 122,551)
Age (years)	50 (46–57)
Sex (male %)	78,390 (64.0%)
Body mass index (kg/m^2^)	23.9 ± 2.8
Systolic blood pressure (mmHg)	125.5 ± 16.4
Diastolic blood pressure (mmHg)	78.8 ± 10.8
Fasting glucose (mg/dL)	96.7 ± 26.2
Total cholesterol (mg/dL)	197.2 ± 35.9
Hypertension	62,641 (51.1%)
Diabetes	14,573 (11.9%)
Dyslipidemia	29,193 (23.8%)
Charlson comorbidity index category	
0	81,851 (66.8%)
1	28,076 (22.9%)
2	8,474 (6.9%)
≥3	4,150 (3.4%)
Current smoker	29,107 (23.8%)
Alcohol consumption	59,018 (48.2%)
Regular exercise	12,329 (10.1%)
Income (lower 10%)	30,869 (25.2%)
Periodontal disease	60,774 (49.6%)
Number of dental caries	
0	100,395 (81.9%)
1–4	18,814 (15.4%)
≥5	3,342 (2.7%)
Number of missing teeth	
0	93,657 (76.4%)
1–4	21,589 (17.6%)
≥5	7,305 (6.0%)
Gum bleeding	98,803 (74.0%)
Tooth brush (time/day)	
0–1	16,768 (13.7%)
2	50,646 (41.3%)
≥3	55,137 (45.0%)
Professional dental cleaning ≥ 1/year	31,903 (26.0%)

Data except ages are expressed as mean ± SD or n (%). Ages are expressed as medians with interquartile ranges.

### Oral health and risk of pneumonia

**Figure 2:**
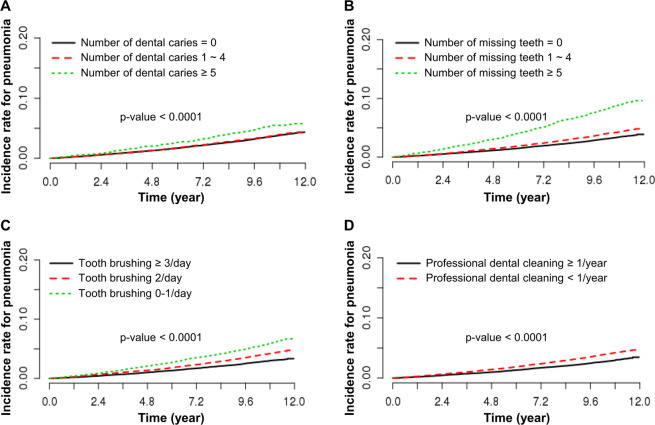
Kaplan-Meier curves for the incidence of pneumonia associated with oral health status and oral hygiene behaviors. (**A**) Number of dental caries, (**B**) number of missing teeth, (**C**) frequency of tooth brushing, and (**D**) frequency of professional dental cleaning. p-value for the log-rank test.

A total of 4,681 pneumonia cases per Def. 3 (3.8%) were identified over a median follow-up period of 10.6 ± 1.1 years. Figure 2 shows the incidence rate of pneumonia according to oral health status and oral hygiene behaviors. The risk of pneumonia was higher in groups with a higher number of dental caries and missing teeth. In contrast, the risk of pneumonia was lower in the frequent tooth brushing group and the regular professional dental cleaning group. There was no significant difference in the risk of pneumonia between groups with and without periodontal disease (p = 0.1904). The number of dental caries and missing teeth, and the frequency of tooth brushing and professional dental cleaning, were associated with the incidence of pneumonia according to multivariable-adjusted Cox proportional hazard models with Def. 3 (Table 2). The risk of pneumonia was significantly higher in the group with a higher number of dental caries (adjusted HR 1.265, 95% CI: 1.086–1.473; p = 0.0025) and the group with more missing teeth (adjusted HR 1.218, 95% CI: 1.113–1.332; p < 0.0001). The risk of pneumonia decreased significantly in the frequent tooth brushing group (adjusted HR 0.853,; 95% CI, 0.786–0.926; p = 0.0001) and in the regular professional dental cleaning group (adjusted HR 0.920,; 95% CI, −0.855–0.990; p = 0.0255). In addition, most of the results using the Fine and Gray model for competing risk were consistent with our main finding (Supplementary Table S8).

**Table 2:** Hazard ratio and 95% confidence interval for incidence of pneumonia (Def. 3) according to oral health status and oral hygiene behaviors (n = 122,551).

	Events (n)	Follow-up duration (person-years)	Incidence rate (per 1,000 person-years)	HR (95% CI)			
				Unadjusted	p-value	Adjusted*	p-value
**Periodontal disease**							
Absent	2,403	646300.0	3.7	1		1	
Present	2,278	636424.5	3.6	0.962 (0.909, 1.019)	0.1906	0.945 (0.891, 1.003)	0.0607
**Number of dental caries**							
0	3,778	1051655.0	3.6	1		1	
1–4	723	196446.2	3.7	1.025 (0.947, 1.110)	0.5433	1.043 (0.961, 1.131)	0.3141
≥5	180	34623.1	5.2	1.448 (1.247, 1.682)	<0.0001	1.265 (1.086, 1.473)	0.0025
**Number of missing teeth**							
0	3,145	984681.7	3.2	1		1	
1–4	914	224793.0	4.1	1.276 (1.186, 1.374)	<0.0001	1.100 (1.021, 1.186)	0.0126
≥5	622	73249.74	8.5	2.682 (2.461, 2.923)	<0.0001	1.218 (1.113, 1.332)	<0.0001
**Number of tooth brush (times/day)**							
0–1	966	172128.9	5.6	1		1	
2	2,091	527852.4	4.0	0.704 (0.652, 0.760)	<0.0001	0.896 (0.829, 0.967)	0.0050
≥3	1,624	582743.2	2.8	0.494 (0.456, 0.535)	<0.0001	0.853 (0.786, 0.926)	0.0001
**Professional dental cleaning**							
<1/year	3,740	337251.2	11.1	1		1	
≥1/year	941	945473.2	1.0	0.703 (0.655, 0.756)	<0.0001	0.920 (0.855, 0.990)	0.0255

Values adjusted for age, sex, body mass index, hypertension, diabetes, dyslipidemia, Charlson comorbidity index category, smoking, drinking, exercise, income, periodontal disease, dental caries, missing teeth, gum bleeding, tooth brush, and professional dental cleaning.

### Comparison of hazard ratios and 95% confidence intervals between definitions of pneumonia

**Figure 3:**
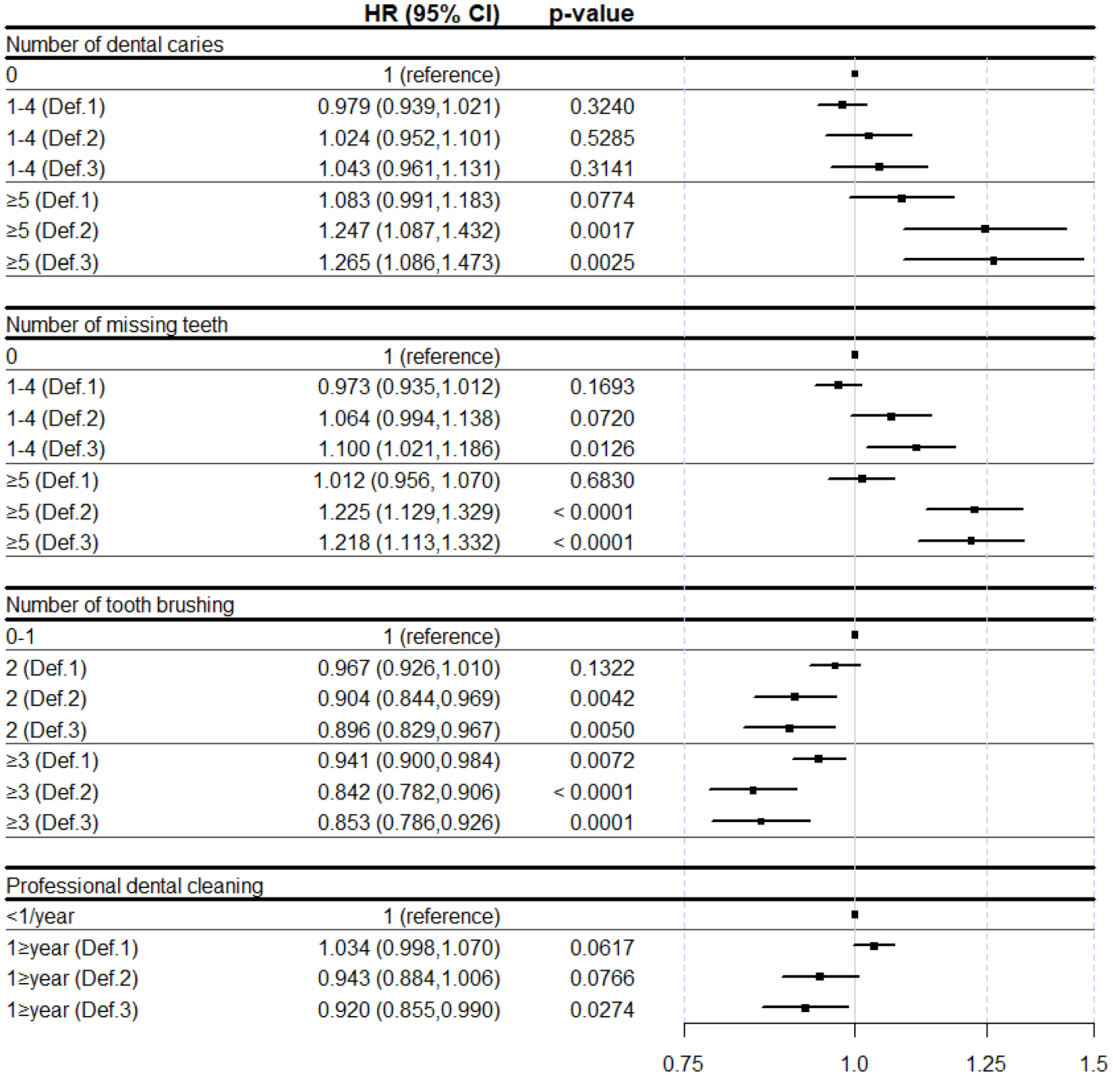
Hazard ratio and 95% confidence interval for incidence of pneumonia according to oral health status and oral hygiene behaviors with different definitions of pneumonia. HR, hazard ratio; CI, confidence interval.

Figure 3 shows the HR and 95% CI for the incidence of pneumonia according to oral health status and oral hygiene behaviors, with different definitions of pneumonia (Def. 1-Def. 3). With Def. 2 and Def. 3, it showed a prominent and significantly adjusted HR and 95% CI compared to Def. 1. The adjusted HR and 95% CI for the incidence of pneumonia with other definitions according to oral health status and oral hygiene behaviors are shown in Supplementary Tables S9–S11.

### Subgroup analysis according to age and comorbidities

**Figure 4:**
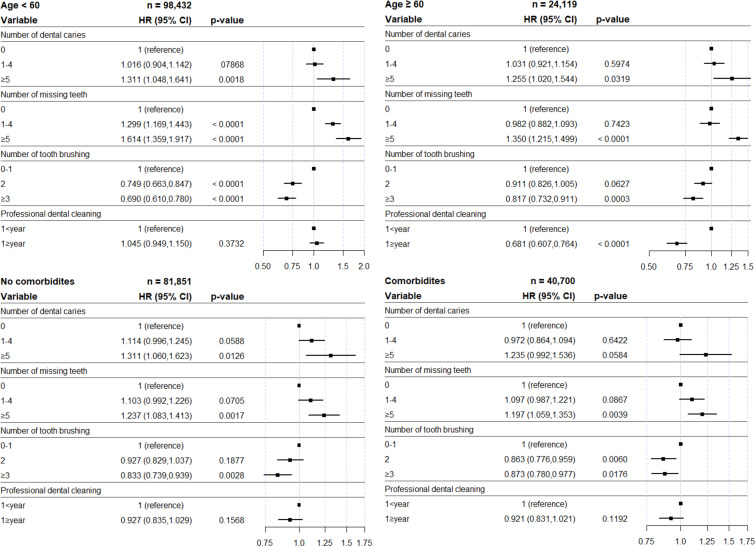
Hazard ratio and 95% confidence interval for incidence of pneumonia in subgroup analysis according to oral health status and oral hygiene behaviors. HR, hazard ratio; CI, confidence interval.

Figure 4 shows the subgroup analysis for Def. 3 according to age and comorbidities. Regardless of age, the number of dental caries and missing teeth were associated with an increased incidence of pneumonia. In the frequent tooth brushing group under 60 years and the regular professional dental cleaning group over 60 years, the risk of pneumonia significantly decreased. Regardless of the underlying disease, the number of missing teeth and the frequency of tooth brushing were associated with the incidence rate of pneumonia.

## Discussion

This study analyzed data from a nationwide population-based Korean cohort from the NHIS-HealS database with a long-term follow-up period, and described the relationship between oral health and the incidence of pneumonia. Oral health status and oral hygiene behaviors were associated with the incidence of pneumonia in the general population. We found that an increased number of dental caries and missing teeth were associated with a higher incidence of pneumonia, and that better oral hygiene care, such as frequent tooth brushing and regular professional dental cleaning, was associated with a lower incidence of pneumonia (Table 2). The HR and 95% CI remained significant after adjustment for covariates including age, sex, BMI, and CCI category. In particular, the link between oral health and pneumonia was more prominent in pneumonia combined with longer hospitalization (Fig. 3). The adjusted HR and 95% CI for the incidence of pneumonia compared to the number of dental caries and tooth brushing sessions per day with the reference group were significant based on Def. 2. For the number of missing teeth and the presence of professional dental cleaning, the HR and 95% CI were significant for Def. 3. Since length of stay is associated with the pneumonia severity index^24,25^, it could be assumed that oral health is related to the severity of pneumonia.

Several studies have investigated the relationship between oral hygiene and pneumonia. More plaque, the presence of bacteria in saliva, and colonization in the oropharynx appear to be associated with pneumonia in hospitalized and ICU patients^26,27^. Manger *et al*. also reported that pneumonia is associated with dental caries and the presence of oral plaque^28^. Suma *et al*. reported that high levels of tooth loss may indicate an increased risk of mortality from pneumonia in the general population^29^. In this study, oral health issues such as dental caries and missing teeth remained significant after adjusting for possible covariates. In addition, Nasiriani *et al*. reported that tooth brushing twice daily in ICU patients can reduce the incidence of ventilator-associated pneumonia^30^. Our results describe that better oral hygiene management, including tooth brushing and professional dental cleaning, remained significant factors after adjusting for covariates.

One remarkable finding in this study was that the association between periodontal disease and pneumonia was not statistically significant. Indeed, in several studies, the results between these two factors were inconsistent. Some epidemiologic studies found no clear relationship between periodontal hygiene and acute respiratory disease^31,32^. However, some studies have reported the association between periodontal disease and pneumonia^33–35^, and Yang *et al*. reported that patients with periodontal treatment exhibited a decreased risk of pneumonia^36^. In this study, NHIS-HealS data did not include the severity and treatment of periodontal disease; therefore, the two factors could potentially be found to be unrelated. Besides, the diagnosis and classification of periodontal disease is the summation of information from medical and dental histories, combined with the findings of clinical and radiographic examinations^37^. Therefore, it is difficult to perform an accurate analysis based simply on information about the presence of periodontal disease obtained only by dental examination, and further research may be necessary.

We performed subgroup analysis, categorized by age and comorbidities in Fig. 4. Many studies have reported an association between oral health and pneumonia in elderly and ICU patients^6–8^, but we confirmed regardless of age and comorbidities, oral health status, and oral hygiene behaviors, especially frequent tooth brushing, was significantly related to the incidence of pneumonia. In fact, tooth brushing by critically ill patients has been advocated by several studies^30,38^, but based on these results, it can be emphasized to the general population.

Several mechanisms have been proposed to explain these results. First, there can be biological plausibility for a causal relationship between oral health and pulmonary disease. An increase in cytokines induced by inflammation of periodontal disease, which is associated with multiple tooth loss and dental caries^39,40^, may promote systemic inflammation, including the lung^41^. Without effective oral care, initial plaque formation can occur within 48 hours and the clump of oropharyngeal flora, which becomes more colonized by gram-negative pathogens, may be transported to the lung, causing pulmonary infection^42^. Second, tooth loss and dental caries are associated with swallowing^43,44^. The loss of processing efficiency for food increases the risk of aspiration into the airways and changes food selection towards decreased consumption of vegetables and fruits^45,46^, causing malnutrition, which is independently associated with pneumonia^47^.

Some limitations should be acknowledged in this study. First, there could be inconsistencies in the diagnostic criteria between dental specialties and general practitioners. In this study, the presence of periodontal diseases was not determined by objective measures such as radiographic examination^37^. In addition, there was a lack of information regarding the severity and treatment of periodontal disease. Second, the number of tooth brushing sessions per day and professional dental cleaning were evaluated using self-reporting questionnaires. Third, there was a possibility that unadjusted covariates could not be excluded. Participants with better oral status and hygiene may have better lifestyles, which can reduce the risk of pneumonia. Finally, there was a possibility that the state of variables would change, which could not guarantee temporality. Future research is required to overcome these limitations.

In conclusion, the increased number of dental caries and missing teeth was associated with an increased incidence of pneumonia. In contrast, improved oral hygiene care, such as frequent tooth brushing and regular professional dental cleaning, was associated with a reduced incidence of pneumonia.

## Supplementary information

Supplementary information.

## References

[1] Henig O, Kaye KS (2017). Bacterial Pneumonia in Older Adults. Infect. Dis. Clin. North Am..

[2] Wise MP, Williams DW, Lewis MA, Thomas JG, Frost PJ (2008). Impact of poor dental health on pneumonia. Eur. Respir. J..

[3] Terpenning M (2005). Geriatric oral health and pneumonia risk. Clin. Infect. Dis..

[4] Terpenning MS (2001). Aspiration pneumonia: dental and oral risk factors in an older veteran population. J. Am. Geriat. Soc..

[5] Paju S, Scannapieco FA (2007). Oral biofilms, periodontitis, and pulmonary infections. Oral Dis..

[6] Yoneyama T, Yoshida M, Matsui T, Sasaki H (1999). Oral care and pneumonia. Oral Care Working Group. Lancet.

[7] Genuit T, Bochicchio G, Napolitano LM, McCarter RJ, Roghman MC (2001). Prophylactic chlorhexidine oral rinse decreases ventilator-associated pneumonia in surgical ICU patients. Surg. Infect..

[8] Mori H (2006). Oral care reduces incidence of ventilator-associated pneumonia in ICU populations. Intensive Care Med..

[9] Jepsen S (2017). Prevention and control of dental caries and periodontal diseases at individual and population level: consensus report of group 3 of joint EFP/ORCA workshop on the boundaries between caries and periodontal diseases. J. Clin. Periodontol..

[10] Chapple IL (2015). Primary prevention of periodontitis: managing gingivitis. J. Clin. Periodontol..

[11] van der Weijden F, Slot DE (2011). Oral hygiene in the prevention of periodontal diseases: the evidence. Periodontology 2000.

[12] Axelsson P, Nystrom B, Lindhe J (2004). The long-term effect of a plaque control program on tooth mortality, caries and periodontal disease in adults. Results after 30 years of maintenance. J. Clin. Periodontol..

[13] Davies GM, Davies RM (2008). Delivering better oral health–an evidence-based toolkit for prevention: a review. Dent. Update.

[14] Seong SC (2017). Cohort profile: the National Health Insurance Service-National Health Screening Cohort (NHIS-HEALS) in Korea. BMJ Open.

[15] *National Health Insurance Sharing Service*, https://nhiss.nhis.or.kr/bd/ab/bdaba000eng.do (2020).

[16] Song TJ, Kim J (2019). Risk of post-stroke pneumonia with proton pump inhibitors, H2 receptor antagonists and mucoprotective agents: A retrospective nationwide cohort study. PloS ONE.

[17] Kim B, Kim J, Jo YH, Lee JH, Hwang JE (2019). The change in age distribution of CAP population in Korea with an estimation of clinical implications of increasing age threshold of current CURB65 and CRB65 scoring system. PloS ONE.

[18] Kim JA (2018). Impact of Visit-to-Visit Fasting Plasma Glucose Variability on the Development of Type 2 Diabetes: A Nationwide Population-Based Cohort Study. Diabetes Care.

[19] Kim, M. K. *et al*. Effects of Variability in Blood Pressure, Glucose, and Cholesterol Concentrations, and Body Mass Index on End-Stage Renal Disease in the General Population of Korea. *J. Clin. Med*. **8**, 10.3390/jcm8050755 (2019). 10.3390/jcm8050755 PMC6571839 31137866

[20] Kim MK (2017). Cholesterol variability and the risk of mortality, myocardial infarction, and stroke: a nationwide population-based study. Eur. Heart J..

[21] Kim KH (2010). [Comparative study on three algorithms of the ICD-10 Charlson comorbidity index with myocardial infarction patients]. J. Prev. Med. Public Health.

[22] de Groot V, Beckerman H, Lankhorst GJ, Bouter LM (2003). How to measure comorbidity. a critical review of available methods. J. Clin. Epidemiol..

[23] Fine JP, Gray RJ (1999). A Proportional Hazards Model for the Subdistribution of a Competing. Risk. J. Am. Stat. Assoc..

[24] Menendez R, Ferrando D, Valles JM, Martinez E, Perpina M (2001). Initial risk class and length of hospital stay in community-acquired pneumonia. Eur. Respir. J..

[25] Garau J (2008). Factors impacting on length of stay and mortality of community-acquired pneumonia. Clin. Microbiol. Infect..

[26] El-Solh AA (2004). Colonization of dental plaques: a reservoir of respiratory pathogens for hospital-acquired pneumonia in institutionalized elders. Chest.

[27] Bonten MJ (1996). Risk factors for pneumonia, and colonization of respiratory tract and stomach in mechanically ventilated ICU patients. *Am*. J. Respir. Crit. Care Med..

[28] Manger D (2017). Evidence summary: the relationship between oral health and pulmonary disease. Brit. Dent. J..

[29] Suma S (2018). Tooth loss and pneumonia mortality: A cohort study of Japanese dentists. PloS ONE.

[30] Nasiriani K, Torki F, Jarahzadeh MH, Rashidi Maybodi F (2016). The Effect of Brushing with a Soft Toothbrush and Distilled Water on the Incidence of Ventilator-Associated Pneumonia in the Intensive Care Unit. Tanaffos.

[31] Scannapieco FA, Papandonatos GD, Dunford RG (1998). Associations between oral conditions and respiratory disease in a national sample survey population. Ann. Periodontol..

[32] Treloar DM, Stechmiller JK (1995). Use of a clinical assessment tool for orally intubated patients. Am. J. Crit. Care.

[33] Iwasaki M (2018). Periodontal disease and pneumonia mortality in haemodialysis patients: A 7-year cohort study. J. Clin. Periodontol..

[34] de Melo Neto JP (2013). Periodontal infections and community-acquired pneumonia: a case-control study. Eur. J. Clin. Microbiol. Infect. Dis..

[35] Gomes-Filho IS (2014). Influence of periodontitis in the development of nosocomial pneumonia: a case control study. Journal of periodontology.

[36] Yang L-C (2020). The Association of Periodontal Treatment and Decreased Pneumonia: A Nationwide Population-Based Cohort Study. Int. J. Environ. Res. Public Health.

[37] Preshaw PM (2015). Detection and diagnosis of periodontal conditions amenable to prevention. BMC Oral Health.

[38] Ames NJ (2011). Evidence to support tooth brushing in critically ill patients. Am. J. Crit. Care.

[39] Merchant AT (2012). Periodontitis and dental caries occur together. J. Evid. Based Dent. Pract..

[40] Nilsson H, Sanmartin Berglund J, Renvert S (2019). Longitudinal evaluation of periodontitis and tooth loss among older adults. J. Clin. Periodontol..

[41] Scannapieco FA, Wang B, Shiau HJ (2001). Oral bacteria and respiratory infection: effects on respiratory pathogen adhesion and epithelial cell proinflammatory cytokine production. Ann. Periodontol..

[42] Scannapieco FA (1999). Role of oral bacteria in respiratory infection. J. Periodontol..

[43] Okamoto N (2012). Relationship between swallowing problems and tooth loss in community-dwelling independent elderly adults: the Fujiwara-kyo study. J. Am. Geriat. Soc..

[44] Furuta M, Yamashita Y (2013). Oral Health and Swallowing Problems. Curr. Phys. Med. Rehabil. Rep..

[45] Quandt SA (2010). Food avoidance and food modification practices of older rural adults: association with oral health status and implications for service provision. Gerontologist.

[46] Savoca MR (2010). Association between dietary quality of rural older adults and self-reported food avoidance and food modification due to oral health problems. J. Am. Geriat. Soc..

[47] Loeb M, High K (2005). The effect of malnutrition on risk and outcome of community-acquired pneumonia. Respir. Care Clin. N. Am..

