# Supplementary material for: Association between oral health and incidence of pneumonia: a population-based cohort study from Korea
Source: Sci Rep. 2020 Jun 12;10:9576. doi: 10.1038/s41598-020-66312-2 (PMC7293333; doi:10.1038/s41598-020-66312-2)

**Association between oral health and incidence of pneumonia: a population-based cohort study from Korea**

**Minkook Son^1^, Sangyong Jo^2^, Ji Sung Lee^3^, and Dong Hyun Lee^4*^**

**Author Affiliations**

^1^Department of Biomedical Science and Engineering, Gwangju Institute of Science and Technology, Gwangju, Korea

^2^Department of Internal Medicine, Air Force 3rd Flying Training Wing, Sacheon, Korea

^3^Clinical Research Center, Asan Medical Center, Ulsan University College of Medicine, Seoul, Korea

^4^Departement of Pulmonology and Critical Care Medicine, Dong-A University, College of Medicine, Busan, Korea

***Corresponding Author**

Dong Hyun Lee, MD, PhD

E-mail: rvot@daum.net, icu1@dau.ac.kr

**Supplementary materials**

Supplementary Table S1. Baseline characteristics of the study participants (with Def. 1).

Supplementary Table S2. Baseline characteristics of the study participants (with Def. 2).

Supplementary Table S3. Comparison of study participants according to the presence of periodontal disease.

Supplementary Table S4. Comparison of study participants according to the number of dental caries.

Supplementary Table S5. Comparison of study participants according to the number of missing teeth.

Supplementary Table S6. Comparison of study participants according to the number of tooth brushing sessions per day.

Supplementary Table S7. Comparison of study participants according to the frequency of professional dental cleaning.

Supplementary Table S8. Hazard ratio and 95% confidence interval for incidence of pneumonia (Def. 3) according to oral health status and oral hygiene behaviors using the Fine and Gray model for competing risk (n = 122,551).

Supplementary Table S9. Hazard ratio and 95% confidence interval for incidence of pneumonia (Def. 1) according to oral health status and oral hygiene behaviors (n = 119,254).

Supplementary Table S10. Hazard ratio and 95% confidence interval for incidence of pneumonia (Def. 2) according to oral health status and oral hygiene behaviors (n = 122,399).

Supplementary Table S11. Hazard ratio and 95% confidence interval for incidence of pneumonia according to oral health status and oral hygiene behaviors (n = 116,245).

Supplementary Figure S1. Definition of pneumonia.

Supplementary Figure S2. Description of the study period.

**Supplementary Table S1. Baseline characteristics of the study participants (with Def. 1).**

| **Characteristics** | **Total**  **(n = 119,254)** |
| --- | --- |
| Age (years) | 50 (46-57) |
| Sex (male %) | 78,568 (64.2%) |
| Body mass index (kg/m^2^) | 23.9 ± 2.8 |
| Systolic blood pressure (mmHg) | 125.5 ± 16.4 |
| Diastolic blood pressure (mmHg) | 78.8 ± 10.8 |
| Fasting glucose (mg/dL) | 96.7 ± 26.2 |
| Total cholesterol (mg/dL) | 197.2 ± 35.9 |
| Hypertension | 60,937 (51.1%) |
| Diabetes | 14,146 (11.9%) |
| Dyslipidemia | 28,389 (23.8%) |
| Charlson comorbidity index category |  |
| 0 | 80,472 (67.5%) |
| 1 | 26,913 (22.6%) |
| 2 | 7,987 (6.7%) |
| ≥ 3 | 3,882 (3.2%) |
| Current smoker | 28,532 (23.9%) |
| Alcohol consumption | 57,742 (48.4%) |
| Regular exercise | 12,004 (10.1%) |
| Income (lower 10%) | 30,043 (25.2%) |
| Periodontal disease | 59,229 (49.7%) |
| Number of dental caries |  |
| 0 | 97,638 (81.9%) |
| 1-4 | 18,351 (15.4%) |
| ≥ 5 | 3,265 (2.7%) |
| Number of missing teeth |  |
| 0 | 91,110 (76.4%) |
| 1-4 | 21,049 (17.6%) |
| ≥ 5 | 7,095 (6.0%) |
| Gum bleeding | 96,046 (80.5%) |
| Tooth brush (time/day) |  |
| 0-1 | 16,341 (13.7%) |
| 2 | 49,202 (41.3%) |
| ≥ 3 | 53,711 (45.0%) |
| Professional dental cleaning ≥ 1/year | 31,081 (26.1%) |

**Supplementary Table S2. Baseline characteristics of the study participants (with Def. 2).**

| **Characteristics** | **Total**  **(n = 122,399)** |
| --- | --- |
| Age (years) | 50 (46-57) |
| Sex (male %) | 78,302 (64.0%) |
| Body mass index (kg/m^2^) | 23.9 ± 2.8 |
| Systolic blood pressure (mmHg) | 125.5 ± 16.4 |
| Diastolic blood pressure (mmHg) | 78.8 ± 10.8 |
| Fasting glucose (mg/dL) | 96.7 ± 26.2 |
| Total cholesterol (mg/dL) | 197.2 ± 35.9 |
| Hypertension | 62,548 (51.1%) |
| Diabetes | 14,548 (11.9%) |
| Dyslipidemia | 29,164 (23.8%) |
| Charlson comorbidity index category |  |
| 0 | 81,814 (66.8%) |
| 1 | 28,011 (22.9%) |
| 2 | 8,442 (6.9%) |
| ≥ 3 | 4,132 (3.4%) |
| Current smoker | 29,077 (23.8%) |
| Alcohol consumption | 58,958 (48.2%) |
| Regular exercise | 12,310 (10.1%) |
| Income (lower 10%) | 30,826 (25.2%) |
| Periodontal disease | 60,697 (49.6%) |
| Number of dental caries |  |
| 0 | 100,267 (81.9%) |
| 1-4 | 18,797 (15.4%) |
| ≥ 5 | 3,335 (2.7%) |
| Number of missing teeth |  |
| 0 | 93,548 (76.4%) |
| 1-4 | 21,559 (17.6%) |
| ≥ 5 | 7,292 (6.0%) |
| Gum bleeding | 98,678 (74.0%) |
| Tooth brush (time/day) |  |
| 0-1 | 16,743 (13.7%) |
| 2 | 50,578 (41.3%) |
| ≥ 3 | 55,078 (45.0%) |
| Professional dental cleaning ≥ 1/year | 31,866 (26.0%) |

**Supplementary Table S3. Comparison of study participants according to the presence of periodontal disease.**

|  | **Presence of periodontal disease** | |
| --- | --- | --- |
| **Characteristics** | **No**  **(n = 61,777)** | **Yes**  **(n = 60,774)** |
| Age (years) | 50 (46-57) | 50 (46-57) |
| Sex (male %) | 37,542 (60.8%) | 40,848 (67.2%) |
| Body mass index (kg/m^2^) | 23.8 ± 2.8 | 23.9 ± 2.8 |
| Systolic blood pressure (mmHg) | 125.2 ± 16.3 | 125.9 ± 16.5 |
| Diastolic blood pressure (mmHg) | 78.5 ± 10.7 | 79.1 ± 10.9 |
| Fasting glucose (mg/dL) | 96.3 ± 25.5 | 97.1± 26.8 |
| Total cholesterol (mg/dL) | 197.2 ± 35.7 | 197.1 ± 36.1 |
| Hypertension | 30,991 (50.2%) | 31,650 (52.1%) |
| Diabetes | 4,692 (7.6%) | 5,089 (8.4%) |
| Dyslipidemia | 14,723 (23.8%) | 14,470 (23.8%) |
| Charlson comorbidity index category |  |  |
| 0 | 40,704 (65.9%) | 41,147 (67.7%) |
| 1 | 14,435 (23.4%) | 13,641 (22.4%) |
| 2 | 4,480 (7.2%) | 3,994 (6.6%) |
| ≥ 3 | 2,158 (3.5%) | 1,992 (3.3%) |
| Current smoker | 12,272 (19.9%) | 16,835 (27.7%) |
| Alcohol consumption | 28,099 (45.5%) | 30,919 (50.9%) |
| Regular exercise | 6,397 (10.4%) | 5,932 (9.8%) |
| Income (lower 10%) | 16,651 (27.0%) | 14,218 (23.4%) |
| Number of dental caries |  |  |
| 0 | 53,886 (87.2%) | 46,509 (76.5%) |
| 1-4 | 7,131 (11.5%) | 11,683 (19.2%) |
| ≥ 5 | 760 (1.3%) | 2,582 (4.3%) |
| Number of missing teeth |  |  |
| 0 | 49,258 (79.7%) | 44,399 (73.1%) |
| 1-4 | 9,146 (14.8%) | 12,443 (20.5%) |
| ≥ 5 | 3,373 (5.5%) | 3,932 (6.4%) |
| Gum bleeding | 51,785 (83.8%) | 47,018 (77.4%) |
| Tooth brush (time/day) |  |  |
| 0-1 | 7,788 (12.6%) | 8,980 (14.8%) |
| 2 | 24,414 (39.5%) | 26,232 (43.1%) |
| ≥ 3 | 29,575 (47.9%) | 25,562 (42.1%) |
| Professional dental  cleaning 1/year | 19,213 (31.1%) | 12,690 (20.9%) |

**Supplementary Table S4. Comparison of the study participants according to the number of dental caries.**

|  | **Number of dental caries** | | |
| --- | --- | --- | --- |
| **Characteristics** | **0**  **(n = 100,395)** | **1-4**  **(n = 18,814)** | **≥ 5**  **(n = 3,342)** |
| Age (years) | 50 (46-57) | 50 (46-56) | 50 (45-57) |
| Sex (male %) | 64,009 (63.8%) | 12,217 (64.9%) | 2,164 (64.8%) |
| BMI (kg/m^2^) | 23.9 ± 2.8 | 23.9 ± 2.9 | 23.6 ± 3.0 |
| Systolic BP (mmHg) | 125.4 ± 16.3 | 126.1 ± 16.5 | 126.2 ± 17.3 |
| Diastolic BP (mmHg) | 78.7 ± 10.8 | 79.1 ± 10.8 | 79.4 ± 11.5 |
| Fasting glucose (mg/dL) | 96.7 ± 25.8 | 96.8 ± 26.9 | 97.8 ± 31.8 |
| Total cholesterol (mg/dL) | 197.4 ± 35.8 | 196.5 ± 36.1 | 193.8 ± 36.8 |
| Hypertension | 50,905 (50.7%) | 9,924 (52.8%) | 1,812 (54.2%) |
| Diabetes | 8,030 (8.0%) | 1,489 (7.9%) | 262 (7.8%) |
| Dyslipidemia | 24,082 (24.0%) | 4,403 (23.4%) | 708 (21.2%) |
| CCI category |  |  |  |
| 0 | 66,546 (66.3%) | 12,975 (69.0%) | 2,330 (69.7%) |
| 1 | 23,269 (23.2%) | 4,094 (21.8%) | 713 (21.3%) |
| 2 | 7,127 (7.1%) | 1,139 (6.0%) | 208 (6.2%) |
| ≥ 3 | 3,453 (3.4%) | 606 (3.2%) | 91 (2.8%) |
| Current smoker | 23,011 (22.9%) | 5,107 (27.1%) | 989 (29.6%) |
| Alcohol consumption | 47,982 (47.8%) | 9,366 (49.8%) | 1,670 (50.0%) |
| Regular exercise | 10,302 (10.3%) | 1,744 (9.3%) | 283 (8.5%) |
| Income (lower 10%) | 26,303 (26.2%) | 3,958 (21.0%) | 608 (18.2%) |
| Periodontal disease | 46,509 (46.3%) | 11,683 (62.1%) | 2,582 (77.3%) |
| Number of missing teeth |  |  |  |
| 0 | 79,412(79.1%) | 12,310 (65.4%) | 1,935 (57.9%) |
| 1-4 | 15,751 (15.7%) | 4,953 (26.3%) | 885 (26.5%) |
| ≥ 5 | 5,232 (5.2%) | 1,551 (8.3%) | 522 (15.6%) |
| Gum bleeding | 81,228 (80.9%) | 14,958 (79.5%) | 2,617 (78.3%) |
| Tooth brush (time/day) |  |  |  |
| 0-1 | 13,358 (13.3%) | 2,891 (15.4%) | 519 (15.5%) |
| 2 | 40,906 (40.8%) | 8,212 (43.6%) | 1,528 (45.7%) |
| ≥ 3 | 46,131 (45.9%) | 7,711 (41.0%) | 1,295 (38.8%) |
| Professional dental  cleaning ≥ 1/year | 27,415(27.3%) | 3,949 (21.0%) | 539 (16.1%) |

BMI, body mass index; CCI, Charlson comorbidity index.

**Supplementary Table S5. Comparison of study participants according to the number of missing teeth.**

|  | **Number of missing teeth** | | |
| --- | --- | --- | --- |
| **Characteristics** | **0**  **(n = 93,657)** | **1-4**  **(n = 21,589)** | **≥ 5**  **(n = 7,305)** |
| Age (years) | 50 (46-56) | 51 (46-58) | 58 (51-66) |
| Sex (male %) | 59,257 (63.3%) | 14,697 (68.1%) | 4,436 (60.7%) |
| BMI (kg/m^2^) | 23.9 ± 2.8 | 24.0 ± 2.8 | 23.7 ± 3.1 |
| Systolic BP (mmHg) | 124.9 ± 16.2 | 127.0 ± 16.6 | 129.4 ± 17.9 |
| Diastolic BP (mmHg) | 78.5 ± 10.8 | 79.6 ± 10.8 | 79.8 ± 11.1 |
| Fasting glucose (mg/dL) | 96.1 ± 25.2 | 98.1 ± 28.0 | 100.1 ± 31.7 |
| Total cholesterol (mg/dL) | 197.2 ± 35.8 | 197.4 ± 35.9 | 196.1 ± 37.4 |
| Hypertension | 46,067 (49.2%) | 12,034 (55.7%) | 4,540 (62.2%) |
| Diabetes | 6,900 (7.4%) | 1,976 (9.2%) | 905 (12.4%) |
| Dyslipidemia | 22,056 (23.6%) | 5,322 (24.7%) | 1,815 (24.9%) |
| CCI category |  |  |  |
| 0 | 62,758 (67.0%) | 14,556 (67.4%) | 4,537 (62.1%) |
| 1 | 21,479 (22.9%) | 4,822 (22.4%) | 1,775 (24.3%) |
| 2 | 6,382 (6.8%) | 1,454 (6.7%) | 638 (8.7%) |
| ≥ 3 | 3,038 (3.3%) | 757 (3.5%) | 355 (4.9%) |
| Current smoker | 20,822 (22.2%) | 6,247 (28.9%) | 2,038 (27.9%) |
| Alcohol consumption | 44,976 (48.0%) | 10,937 (50.7%) | 3,105 (42.5%) |
| Regular exercise | 9,386 (10.0%) | 2,162 (10.0%) | 781 (10.7%) |
| Income (lower 10%) | 25,290 (27.0%) | 4,518 (20.9%) | 1,061 (14.5%) |
| Periodontal disease | 44,399 (47.4%) | 12,443 (57.6%) | 3,932 (53.8%) |
| Number of dental caries |  |  |  |
| 0 | 79,412 (84.8%) | 15,751 (73.0%) | 5,232 (71.6%) |
| 1-4 | 12,310 (13.1%) | 4,953 (22.9%) | 1,551 (21.2%) |
| ≥ 5 | 1,935 (2.1%) | 885 (4.1%) | 522 (7.2%) |
| Gum bleeding | 75,905 (81.1%) | 17,046 (79.0%) | 5,852 (80.1%) |
| Tooth brush (time/day) |  |  |  |
| 0-1 | 11,854 (12.7%) | 3,366 (15.6%) | 1,548 (21.2%) |
| 2 | 37,788 (40.3%) | 9,450 (43.8%) | 3,408 (46.6%) |
| ≥ 3 | 44,015 (47.0%) | 8,773 (40.6%) | 2,349 (32.2%) |
| Professional dental cleaning ≥ 1/year | 25,109 (26.8%) | 5,466 (25.3%) | 1,328 (18.2%) |

BMI, body mass index; CCI, Charlson comorbidity index.

**Supplementary Table S6. Comparison of study participants according to the number of tooth brushing sessions per day.**

|  | **Number of tooth brush** | | |
| --- | --- | --- | --- |
| **Characteristics** | **0-1**  **(n = 16,768)** | **2**  **(n = 50,646)** | **≥ 3**  **(n = 55,137)** |
| Age (years) | 53 (48-61) | 52 (46-58) | 49 (45-55) |
| Sex (male %) | 11,774 (70.2%) | 30,567 (60.4%) | 36,049 (65.4%) |
| BMI (kg/m^2^) | 23.9 ± 2.9 | 24.0 ± 2.9 | 23.8 ± 2.7 |
| Systolic BP (mmHg) | 127.4 ± 16.8 | 125.9 ± 16.7 | 124.6 ± 16.0 |
| Diastolic BP (mmHg) | 79.5 ± 10.8 | 78.8 ± 10.9 | 78.6 ± 10.7 |
| Fasting glucose (mg/dL) | 99.1 ± 29.7 | 97.1 ± 26.4 | 95.7 ± 24.7 |
| Total cholesterol (mg/dL) | 196.8 ± 36.4 | 197.8 ± 36.3 | 196.7 ± 35.3 |
| Hypertension | 9,482 (56.6%) | 26,464 (52.3%) | 26,695 (48.4%) |
| Diabetes | 1,649 (9.8%) | 4,289 (8.5%) | 3,843 (7.0%) |
| Dyslipidemia | 4,152 (24.8%) | 12,414 (24.5%) | 12,627 (22.9%) |
| CCI category |  |  |  |
| 0 | 10,781 (64.3%) | 33,011 (65.2%) | 38,059 (69.0%) |
| 1 | 3,948 (23.5%) | 12,041 (23.8%) | 12,087 (21.9%) |
| 2 | 1,338 (8.0%) | 3,755 (7.4%) | 3,381 (6.1%) |
| ≥ 3 | 701 (4.2%) | 1,839 (3.6%) | 1,610 (3.0%) |
| Current smoker | 4,821 (28.8%) | 12,451 (24.6%) | 11,835 (21.5%) |
| Alcohol consumption | 8,417 (50.2%) | 23,379 (46.2%) | 27,222 (49.4%) |
| Regular exercise | 1,524 (9.1%) | 4,859 (9.6%) | 5,946 (10.8%) |
| Income (lower 10%) | 3,337 (19.9%) | 10,781 (21.3%) | 16,751 (30.4%) |
| Periodontal disease | 8,980 (53.6%) | 26,232 (51.8%) | 25,562 (46.4%) |
| Number of dental caries |  |  |  |
| 0 | 13,358 (79.7%) | 40,906 (80.8%) | 46,131 (83.7%) |
| 1-4 | 2,891 (17.2%) | 8,212 (16.2%) | 7,711 (14.0%) |
| ≥ 5 | 519 (3.1%) | 1,528 (3.0%) | 1,295 (2.3%) |
| Number of missing teeth |  |  |  |
| 0 | 11,854 (70.7%) | 37,788 (74.6%) | 44,015 (79.8%) |
| 1-4 | 3,366 (20.1%) | 9,450 (18.7%) | 8,773 (15.9%) |
| ≥ 5 | 1,548 (9.2%) | 3,408 (6.7%) | 2,349 (4.3%) |
| Gum bleeding | 13,370 (79.7%) | 40,381 (79.7%) | 45,052 (81.7%) |
| Professional dental cleaning ≥ 1/year | 3,591 (21.4%) | 11,792 (23.3%) | 16,520 (30.0%) |

BMI, body mass index; CCI, Charlson comorbidity index.

**Supplementary Table S7. Comparison of study participants according to the frequency of professional dental cleaning.**

|  | **Frequency of professional dental cleaning** | |
| --- | --- | --- |
| **Characteristics** | **< 1/year**  **(n = 90,648)** | **≥ 1/year**  **(n = 31,903)** |
| Age (years) | 51 (46-58) | 50 (46-55) |
| Sex (male %) | 56,129 (61.9%) | 22,261 (69.8%) |
| BMI (kg/m^2^) | 23.9 ± 2.8 | 24.0 ± 2.7 |
| Systolic blood pressure (mmHg) | 125.8 ± 16.6 | 124.7 ± 15.8 |
| Diastolic blood pressure (mmHg) | 78.8 ± 10.9 | 78.6 ± 10.6 |
| Fasting glucose (mg/dL) | 96.7 ± 26.3 | 96.9 ± 25.8 |
| Total cholesterol (mg/dL) | 196.9 ± 36.0 | 197.8 ± 35.6 |
| Hypertension | 46,987 (51.8%) | 15,654 (49.1%) |
| Diabetes | 7,302 (8.1%) | 2,479 (7.8%) |
| Dyslipidemia | 21,516 (23.7%) | 7,677 (24.1%) |
| CCI category |  |  |
| 0 | 60,397 (66.6%) | 21,454 (67.2%) |
| 1 | 20,806 (23.0%) | 7,270 (22.8%) |
| 2 | 6,322 (7.0%) | 2,152 (6.8%) |
| ≥ 3 | 3,123 (3.4%) | 1,027 (3.2%) |
| Current smoker | 21,050 (23.2%) | 8,057 (25.2%) |
| Alcohol consumption | 42,194 (46.6%) | 16,824 (52.7%) |
| Regular exercise | 9,080 (10.0%) | 3,249 (10.2%) |
| Income (lower 10%) | 20,867 (23.0%) | 10,002 (31.4%) |
| Periodontal disease | 48,084 (53.0%) | 12,690 (39.8%) |
| Number of dental caries |  |  |
| 0 | 72,980 (80.5%) | 27,415 (85.9%) |
| 1-4 | 14,865 (16.4%) | 3,949 (12.4%) |
| ≥ 5 | 2,803 (3.1%) | 539 (1.7%) |
| Number of missing teeth |  |  |
| 0 | 68,548 (75.6%) | 25,109 (78.7%) |
| 1-4 | 16,123 (17.8%) | 5,466 (17.1%) |
| ≥ 5 | 5,977 (6.6%) | 1,328 (4.2%) |
| Gum bleeding | 73,879 (81.5%) | 24,924 (78.1%) |
| Tooth brush (time/day) |  |  |
| 0-1 | 13,177 (14.5%) | 3,591 (11.2%) |
| 2 | 38,854 (42.9%) | 11,792 (37.0%) |
| ≥ 3 | 38,617 (42.6%) | 16,520 (51.8%) |

BMI, body mass index; CCI, Charlson comorbidity index.

**Supplementary Table S8. Hazard ratio and 95% confidence interval for incidence of pneumonia (Def. 3) according to oral health status and oral hygiene behaviors using the Fine and Gray model for competing risk (n = 122,551).**

|  | Events (n) | Follow-up duration (person-years) | Incidence rate  (per 1,000  person-years) | **HR (95% CI)** | | | |
| --- | --- | --- | --- | --- | --- | --- | --- |
|  |  |  |  | **Unadjusted** | **p-value** | **Adjusted^*^** | **p-value** |
| **Periodontal disease** |  |  |  |  |  |  |  |
| Absent | 2,403 | 646300.0 | 3.7 | 1 |  | 1 |  |
| Present | 2,278 | 636424.5 | 3.6 | 0.962 (0.908, 1.018) | 0.1822 | 0.954 (0.900, 1.013) | 0.1236 |
| **Number of dental caries** |  |  |  |  |  |  |  |
| 0 | 3,778 | 1051655.0 | 3.6 | 1 |  | 1 |  |
| 1-4 | 723 | 196446.2 | 3.7 | 1.022 (0.944, 1.106) | 0.5960 | 1.030 (0.949, 1.118) | 0.4848 |
| ≥5 | 180 | 34623.1 | 5.2 | 1.438 (1.238, 1.670) | < 0.0001 | 1.229 (1.051, 1.437) | 0.0096 |
| **Number of missing teeth** |  |  |  |  |  |  |  |
| 0 | 3,145 | 984681.7 | 3.2 | 1 |  | 1 |  |
| 1-4 | 914 | 224793.0 | 4.1 | 1.270 (1.180, 1.367) | < 0.0001 | 1.106 (1.026, 1.193) | 0.0087 |
| ≥5 | 622 | 73249.74 | 8.5 | 2.605 (2.390, 2.839) | < 0.0001 | 1.191 (1.086, 1.306) | 0.0002 |
| **Number of tooth brush (times/day)** |  |  |  |  |  |  |  |
| 0-1 | 966 | 172128.9 | 5.6 | 1 |  | 1 |  |
| 2 | 2,091 | 527852.4 | 4.0 | 0.711 (0.659, 0.767) | < 0.0001 | 0.905 (0.838, 0.978) | 0.0119 |
| ≥3 | 1,624 | 582743.2 | 2.8 | 0.502 (0.464, 0.544) | < 0.0001 | 0.864 (0.796, 0.939) | 0.0005 |
| **Professional dental cleaning** |  |  |  |  |  |  |  |
| <1/year | 3,740 | 337251.2 | 11.1 | 1 |  | 1 |  |
| ≥1/year | 941 | 945473.2 | 1.0 | 0.709 (0.660, 0.761) | < 0.0001 | 0.933 (0.867, 1.048) | 0.0628 |

Values adjusted for age, sex, body mass index, hypertension, diabetes, dyslipidemia, Charlson comorbidity index category, smoking, drinking, exercise, income, periodontal disease, dental caries, missing teeth, gum bleeding, tooth brush, and professional dental cleaning.

**Supplementary Table S9. Hazard ratio and 95% confidence interval for incidence of pneumonia (Def. 1) according to oral health status and oral hygiene behaviors (n = 119,254).**

|  | Events (n) | Follow-up duration (person-years) | Incidence rate  (per 1,000  person-years) | **HR (95% CI)** | | | |
| --- | --- | --- | --- | --- | --- | --- | --- |
|  |  |  |  | **Unadjusted** | **p-value** | **Adjusted^*^** | **p-value** |
| **Periodontal disease** |  |  |  |  |  |  |  |
| Absent | 9,246 | 593335.4 | 15.6 | 1 |  | 1 |  |
| Present | 8,758 | 587904.7 | 14.9 | 0.956 (0.928, 0.984) | 0.0023 | 0.976 (0.947, 1.006) | 0.1122 |
| **Number of dental caries** |  |  |  |  |  |  |  |
| 0 | 14,848 | 966891.5 | 15.4 | 1 |  | 1 |  |
| 1-4 | 2,633 | 182260.4 | 14.4 | 0.941 (0.902, 0.980) | 0.0037 | 0.979 (0.939, 1.021) | 0.3240 |
| ≥5 | 523 | 32088.2 | 16.3 | 1.062 (0.973, 1.158) | 0.1784 | 1.083 (0.991, 1.183) | 0.0774 |
| **Number of missing teeth** |  |  |  |  |  |  |  |
| 0 | 13,422 | 906146.2 | 14.8 | 1 |  | 1 |  |
| 1-4 | 3,146 | 208024.5 | 15.1 | 1.022 (0.983, 1.063) | 0.2669 | 0.973 (0.935, 1.012) | 0.1693 |
| ≥5 | 1,436 | 67069.5 | 21.4 | 1.454 (1.377, 1.536) | < 0.0001 | 1.012 (0.956, 1.070) | 0.6830 |
| **Number of tooth brush (times/day)** |  |  |  |  |  |  |  |
| 0-1 | 2,845 | 158134.4 | 18.0 | 1 |  | 1 |  |
| 2 | 7,814 | 483831.7 | 16.2 | 0.896 (0.859, 0.936) | < 0.0001 | 0.967 (0.926, 1.010) | 0.1322 |
| ≥3 | 7,345 | 539274.0 | 13.6 | 0.754 (0.722, 0.787) | < 0.0001 | 0.941 (0.900, 0.984) | 0.0072 |
| **Professional dental cleaning** |  |  |  |  |  |  |  |
| <1/year | 13,533 | 870602.4 | 15.5 | 1 |  | 1 |  |
| ≥1/year | 4,471 | 310637.7 | 14.4 | 0.924 (0.894, 0.956) | < 0.0001 | 1.034 (0.998, 1.070) | 0.0617 |

Values adjusted for age, sex, body mass index, hypertension, diabetes, dyslipidemia, Charlson comorbidity index category, smoking, drinking, exercise, income, periodontal disease, dental caries, missing teeth, gum bleeding, tooth brush, and professional dental cleaning.

**Supplementary Table S10. Hazard ratio and 95% confidence interval for incidence of pneumonia (Def. 2) according to oral health status and oral hygiene behaviors (n = 122,399).**

|  | Events (n) | Follow-up duration (person-years) | Incidence rate  (per 1,000  person-years) | **HR (95% CI)** | | | |
| --- | --- | --- | --- | --- | --- | --- | --- |
|  |  |  |  | **Unadjusted** | **p-value** | **Adjusted^*^** | **p-value** |
| **Periodontal disease** |  |  |  |  |  |  |  |
| Absent | 3,045 | 642906.6 | 4.7 | 1 |  | 1 |  |
| Present | 2,849 | 633457.2 | 4.5 | 0.949 (0.902, 0.999) | 0.0459 | 0.937 (0.889, 0.988) | 0.0162 |
| **Number of dental caries** |  |  |  |  |  |  |  |
| 0 | 4,781 | 1046427.0 | 4.6 | 1 |  | 1 |  |
| 1-4 | 894 | 195575.4 | 4.6 | 1.001 (0.932, 1.075) | 0.9816 | 1.024 (0.952, 1.101) | 0.5285 |
| ≥5 | 219 | 34361.3 | 6.4 | 1.399 (1.222, 1.602) | < 0.0001 | 1.247 (1.087, 1.432) | 0.0017 |
| **Number of missing teeth** |  |  |  |  |  |  |  |
| 0 | 4,021 | 980027.0 | 4.1 | 1 |  | 1 |  |
| 1-4 | 1,116 | 223692.4 | 5.0 | 1.219 (1.141, 1.302) | < 0.0001 | 1.064 (0.994, 1.138) | 0.0720 |
| ≥5 | 757 | 72644.5 | 10.4 | 2.563 (2.371, 2.770) | < 0.0001 | 1.225 (1.129, 1.329) | < 0.0001 |
| **Number of tooth brush (times/day)** |  |  |  |  |  |  |  |
| 0-1 | 1,189 | 171018.3 | 7.0 | 1 |  | 1 |  |
| 2 | 2,648 | 524955.6 | 5.0 | 0.724 (0.676, 0.775) | < 0.0001 | 0.904 (0.844, 0.969) | 0.0042 |
| ≥3 | 2,057 | 580389.2 | 3.5 | 0.507 (0.472, 0.544) | < 0.0001 | 0.842 (0.782, 0.906) | < 0.0001 |
| **Professional dental cleaning** |  |  |  |  |  |  |  |
| <1/year | 4,665 | 335672.2 | 13.9 | 1 |  | 1 |  |
| ≥1/year | 1,229 | 940691.7 | 1.3 | 0.736 (0.691, 0.784) | < 0.0001 | 0.943 (0.884, 1.006) | 0.0766 |

Values adjusted for age, sex, body mass index, hypertension, diabetes, dyslipidemia, Charlson comorbidity index category, smoking, drinking, exercise, income, periodontal disease, dental caries, missing teeth, gum bleeding, tooth brush, and professional dental cleaning.

**Supplementary Table S11. Hazard ratio and 95% confidence interval for incidence of pneumonia according to oral health status and oral hygiene behaviors (n = 116,245).**

|  | Events (n) | Follow-up duration (person-years) | Incidence rate  (per 1,000  person-years) | **HR (95% CI)** | | | |
| --- | --- | --- | --- | --- | --- | --- | --- |
|  |  |  |  | **Unadjusted** | **p-value** | **Adjusted^*^** | **p-value** |
| **Periodontal disease** |  |  |  |  |  |  |  |
| Absent | 11,426 | 561622.1 | 20.3 | 1 |  | 1 |  |
| Present | 10,927 | 557276.8 | 19.6 | 0.964 (0.939, 0.989) | 0.0057 | 0.985 (0.959, 1.012) | 0.2639 |
| **Number of dental caries** |  |  |  |  |  |  |  |
| 0 | 18,424 | 915321.3 | 20.1 | 1 |  | 1 |  |
| 1-4 | 3,276 | 173242.9 | 18.9 | 0.939 (0.905, 0.975) | 0.0010 | 0.973 (0.937, 1.011) | 0.1566 |
| ≥5 | 653 | 30334.7 | 21.5 | 1.069 (0.989, 1.156) | 0.0916 | 1.090 (1.007, 1.180) | 0.0325 |
| **Number of missing teeth** |  |  |  |  |  |  |  |
| 0 | 16,631 | 860315.4 | 19.3 | 1 |  | 1 |  |
| 1-4 | 3,947 | 196843.1 | 20.1 | 1.038 (1.002, 1.074) | 0.0364 | 0.992 (0.957, 1.027) | 0.6379 |
| ≥5 | 1,775 | 61740.4 | 28.7 | 1.490 (1.419, 1.565) | < 0.0001 | 1.045 (0.994, 1.100) | 0.0858 |
| **Number of tooth brush (times/day)** |  |  |  |  |  |  |  |
| 0-1 | 3,525 | 148201.7 | 23.8 | 1 |  | 1 |  |
| 2 | 9,721 | 456761.8 | 21.3 | 0.894 (0.861, 0.930) | < 0.0001 | 0.957 (0.921, 0.995) | 0.0274 |
| ≥3 | 9,107 | 513935.3 | 17.7 | 0.744 (0.716, 0.774) | < 0.0001 | 0.923 (0.887, 0.960) | <0.0001 |
| **Professional dental cleaning** |  |  |  |  |  |  |  |
| <1/year | 16,810 | 823038.6 | 20.4 | 1 |  | 1 |  |
| ≥1/year | 5,543 | 295860.3 | 18.7 | 0.917 (0.889, 0.945) | < 0.0001 | 1.029 (0.997, 1.061) | 0.0725 |

ICD-10 code for pneumonia (J10-J18)

Values adjusted for age, sex, body mass index, hypertension, diabetes, dyslipidemia, Charlson comorbidity index category, smoking, drinking, exercise, income, periodontal disease, dental caries, missing teeth, gum bleeding, tooth brush, and professional dental cleaning.

**Supplementary Figure S1. Definition of pneumonia.**

Def., Definition.


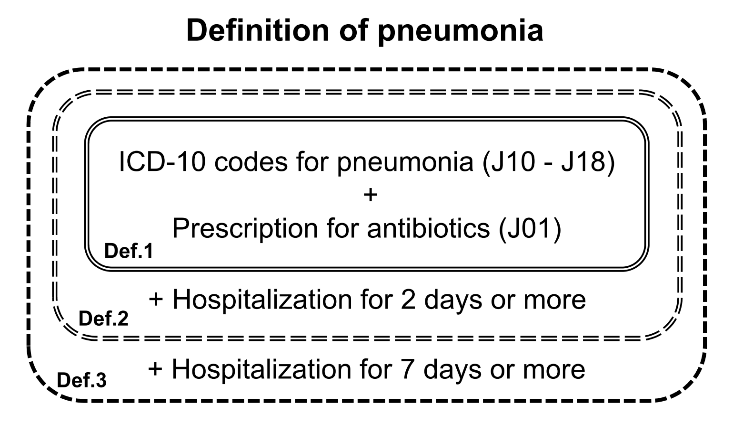


**Supplementary Figure S2. Description of the study period.**


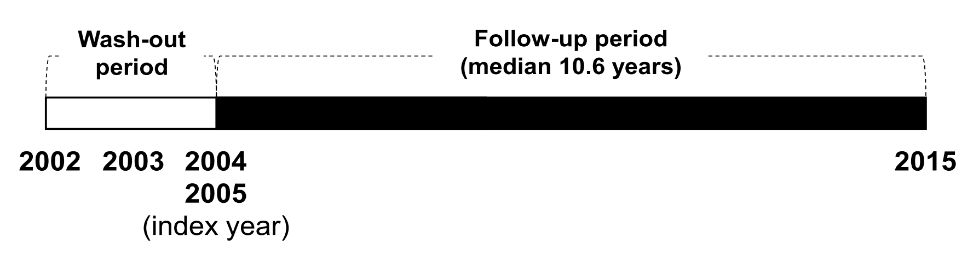

Supplement: Supplementary file 1 — Supplementary information. [file 41598_2020_66312_MOESM1_ESM.docx]
